# Vascular risk factors for idiopathic normal pressure hydrocephalus: a systematic review and meta-analysis

**DOI:** 10.3389/fneur.2023.1220473

**Published:** 2023-08-10

**Authors:** Hanlin Cai, Feng Yang, Hui Gao, Keru Huang, Linyuan Qin, Ruihan Wang, Yi Liu, Liangxue Zhou, Zilong Hao, Dong Zhou, Qin Chen

**Affiliations:** ^1^Department of Neurology, West China Hospital of Sichuan University, Chengdu, China; ^2^Department of Neurosurgery, West China Hospital of Sichuan University, Chengdu, China

**Keywords:** idiopathic normal pressure hydrocephalus, vascular risk factors, hypertension, diabetes mellitus, meta-analysis

## Abstract

**Objective:**

Idiopathic normal-pressure hydrocephalus (iNPH) is a treatable cause of dementia; however, its etiology and pathogenesis remain poorly understood. The objective of this study was to investigate the prevalence and impact of vascular risk factors in patients with iNPH compared to a control cohort to better understand the potential mechanisms and preventive measures.

**Methods:**

We systematically searched PubMed, Web of Science, Embase, and the Cochrane Library (from inception to December 20, 2022) for studies reporting vascular risk factors for the development of iNPH. Pooled odds ratios (ORs) and 95% confidence intervals (CIs) were estimated using random-effects models.

**Results:**

After screening 1,462 articles, 11 case-control studies comprising 1,048 patients with iNPH and 79,668 cognitively unimpaired controls were included in the meta-analysis. Our data showed that hypertension (*N* = 991, OR = 2.30, 95% CI 1.64 to 3.23, *I*^2^= 64.0%), diabetes mellitus (DM) (*N* = 985, OR = 3.12, 95% CI 2.29 to 4.27, *I*^2^= 44.0%), coronary heart disease (CHD; *N* = 880, OR = 2.34, 95% CI 1.33 to 4.12, *I*^2^= 83.1%), and peripheral vascular disease (*N* = 172, OR = 2.77, 95% CI 1.50 to 5.13, *I*^2^= 0.0%) increased the risk for iNPH, while overweight was a possible factor (*N* = 225, OR = 2.01, 95% CI 1.34 to 3.04, *I*^2^= 0.0%) based on the sensitivity analysis. Smoking and alcohol consumption were not associated with iNPH.

**Conclusions:**

Our study suggested that hypertension, DM, CHD, peripheral vascular disease, and overweight were associated with iNPH. These factors might be involved in the pathophysiological mechanisms promoting iNPH. These findings require further investigation in future studies.

**Systematic review registration:**

https://www.crd.york.ac.uk/PROSPERO/, CRD42022383004.

## 1. Introduction

Idiopathic normal-pressure hydrocephalus (iNPH) is a clinical syndrome characterized by cognitive decline, gait disturbance, and urinary incontinence, with ventricular enlargement apparent on brain imaging (1). A recent epidemiological study revealed a prevalence of 0.2% in population aged 70–79 years and ~6% in those 80 years and older (2). As expected, with an aging population, the number of patients with iNPH has been steadily increasing. Currently, iNPH is a treatable cause of dementia; however, it is often underdiagnosed and undertreated (3). In addition, the etiology and pathogenesis of this disease remain poorly understood.

Some observational studies have indicated that about one in four patients with iNPH have vascular risk factors (4). At present, several vascular risk factors for iNPH have been reported, including hypertension (2, 4–15), diabetes mellitus (DM) (4, 6–10, 12–14, 16), hyperlipidemia (4, 7, 13), smoking (4, 6, 7, 10, 13, 14), alcohol use (6, 14), overweight (2, 4, 6, 7), coronary heart disease (CHD) (4, 6, 8–10, 12–14), and peripheral vascular disease (4, 13); some may play an important role in the development of iNPH. However, these findings have been inconsistent (4–6, 8, 10, 13, 14). In addition, most of those studies involved relatively small sample sizes. Therefore, we conducted a systematic review and meta-analysis to investigate the association between vascular risk factors and iNPH for understanding the potential mechanisms and preventive measures.

## 2. Methods

### 2.1. Search strategy

This study was conducted in accordance with the Preferred Reporting Items for Systematic Reviews and Meta-Analyses guidelines (17). We have registered this meta-analysis on PROSPERO (CRD42022383004). We systematically searched the PubMed, Embase, Web of Science, and Cochrane Library databases (from inception to December 20, 2022) for observational studies (cohort, cross-sectional, and case-control studies) to evaluate the association between vascular risk factors and iNPH. The following search strategy was used for these databases, with appropriate modifications, by two investigators (HL. C and F. Y): (“Hydrocephalus, Normal Pressure” OR “Normal Pressure Hydrocephalus” OR “Hakim Syndrome”) AND (“Risk Factors” OR “Hypertension” OR “High blood pressure” OR “Diabetes Mellitus” OR “Diabetes” OR “Hyperlipidemias” OR “Overweight” OR “Obesity” OR “Smoking” OR “Alcohol Drinking”). The reference lists of the included studies were also reviewed.

### 2.2. Inclusion and exclusion criteria

Studies that met the following criteria were included: (1) patients with iNPH with at least one of the features of the Hakim's triad (gait disturbance, cognitive impairment, and urinary problems) and radiologically confirmed ventricular enlargement according to the existing diagnostic criteria (18); (2) study evaluating at least one of the predefined vascular risk factors for iNPH [adapting from INTERHEART (19) and INTERSTROKE (20) study], including hypertension, DM, hyperlipidemia, smoking, alcohol use, overweight, CHD, and peripheral vascular diseases (4); and (3) control group consisted of cognitively unimpaired individuals.

Studies were excluded if they: (1) were reviews, meeting abstracts, editorial materials, or articles not published in English; (2) the odds ratios (ORs) and 95% confidence intervals (CIs) were unextractable; (3) included participants with symptomatic or secondary NPH, including cerebrovascular diseases, head trauma, brain tumors, or infections; and (4) only evaluated patients with asymptomatic ventricular enlargement.

### 2.3. Data extraction and quality assessment

The titles and abstracts were independently screened by HL. C and F. Y, who read the full text and extracted the data. Any discrepancies were resolved through consensus. The following information was extracted from each study using a predesigned data extraction form: name of the first author, publication year, country, sample size, age, sex, study design, matched factors, diagnostic criteria for iNPH, and risk factors investigated. Adjusted data were recorded when studies reported both crude and adjusted ORs. The calculation of the crude OR and interval estimation were based on previously published methods when a specific OR was not provided in the original articles (21). Quality assessments were performed using the Newcastle–Ottawa Scale (NOS) (https://www.ohri.ca/programs/clinical_epidemiology/oxford.asp). A “star system” was devised to evaluate studies, with a focus on three aspects: the selection of the study groups, comparability of the groups, and ascertainment of either the exposure or outcome of interest in the context of case-control or cohort studies, respectively. Two investigators (HL. C and F. Y) independently assessed all eligible studies, and disagreements were resolved through consensus. Articles with 8–9 stars were rated as high quality, 5–7 stars as moderate quality, and 4 stars as low quality (22).

### 2.4. Statistical analysis

A pooled OR with a 95% CI was calculated for patients with iNPH based on possible vascular risk factors. The *I*^2^ test was used to quantify heterogeneity, and an *I*^2^ value >50% was considered to indicate substantial heterogeneity (23). Since clinical heterogeneity between studies was significant, all meta-analyses were conducted using random-effect models (Dersimonian-Laird method). Meta-regression analysis was also conducted based on the mean age, sex, region, year of publication, and sample sizes of certain studies. Sensitivity analysis for potential factors (*N* ≥ 3) was performed by eliminating one study at a time to evaluate the stability of the results and explain the possible sources of heterogeneity. Publication bias was assessed by visually inspecting the funnel plot and statistically examining the results using the Begg's and Egger's tests if more than five studies were synthesized for each factor. *P* < 0.05 was considered statistically significant. All statistical analyses were performed using Stata SE 16.0 (StataCorp., T.X., USA).

## 3. Results

### 3.1. Literature search

**Figure 1:**
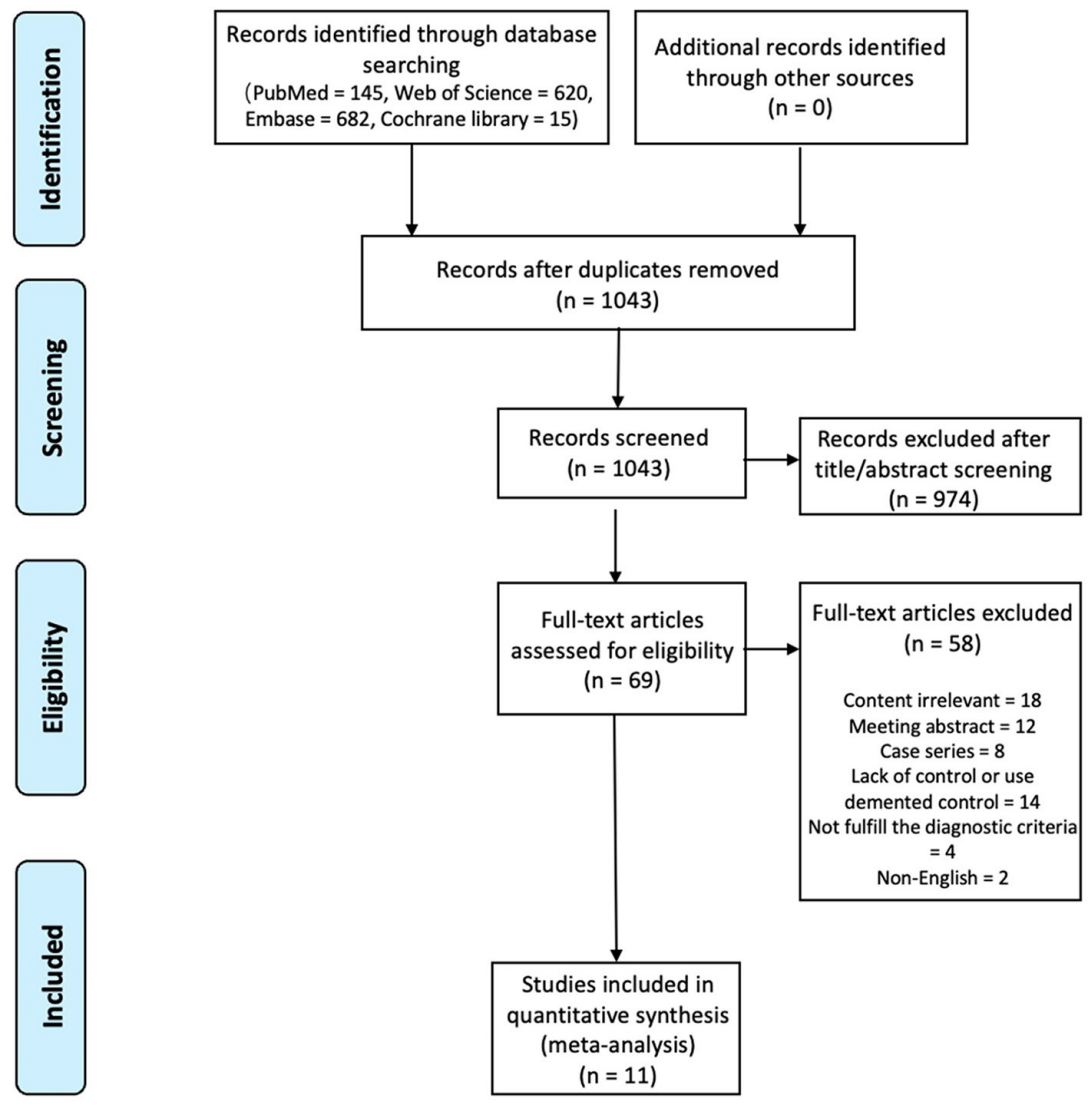
Flowchart of literature processing according to the PRISMA guidelines.

In total, 1,462 publications were retrieved after the initial search. The titles and abstracts of 1,043 studies were screened after removing duplicates, leaving 69 articles that were assessed for eligibility by reading the full text. Fifty-eight records were excluded for several reasons. Finally, 11 studies were included in the meta-analysis (Figure 1).

### 3.2. Characteristics of included studies

Detailed characteristics of the included studies are presented in Table 1. The included studies consisted of 11 case-control studies, which were conducted in the United States, Italy, Germany, Norway, Sweden, Finland, and Japan. Among these studies, 1,048 patients with iNPH (sample size ranging from 12 to 440 with a median of 29) and 79,668 controls were included. For the diagnosis of iNPH, seven studies used the American-European Guideline criteria (18), three used self-defined criteria, and one used the Japanese criteria (24) (Supplementary Table S1). Most patients with iNPH were over 70 years of age. For vascular risk factors, eight vascular risk factors were evaluated, including hypertension, hyperlipidemia, DM, overweight, smoking, alcohol use, CHD, and peripheral vascular disease. The definitions of the vascular risk factors for each included study are summarized in Supplementary Table S2. Ten studies included hypertension, nine studies mentioned DM, seven studies mentioned CHD, and seven studies included these three risk factors. All the included studies were of moderate to high quality. The NOS scores ranged from 6 to 9, with a median NOS score of 8 (Supplementary Table S4).

**Table 1:** The characteristics of included studies in the meta-analysis.

**References**	**Country**	**Study design**	**No. of particpants (iNPH/controls)**	**Age of participants (iNPH/controls)**	**Female of iNPH patients (%)**	**Factors matched**	**iNPH diagnostic criteria**	**Controls**	**Risk factor investigated**
Jacobs (16)	USA	Case–control	33/33	70.27 ± 8.01/70.52 ± 8.18	16/33 (48.5%)	Age	At least one symptom of iNPH triad and hydrocephalic pneumoencephalography or radioisotopic cisternography findings.	Hospital-based controls without neurological disorders	2
Casmiro (6)	Italy	Case–control	17/51	69.65/ 70.25	4/17 (23.5%)	Age, sex	At least one symptom of iNPH triad and hydrocephalic CT findings.	Hospital-based controls (*n* = 17) and population-based controls (*n* = 34)	1, 2, 3, 4
Krauss et al. (7)	Germany	Case–control	65/70	70.8 ± 7.4/ 69.3 ± 5.9	35/65 (53.8%)	Age	Clinical presentation of NPH consisting of gait disturbance with or without dementia and/or urinary incontinence, ventricular enlargement, and the absence of cortical atrophy.	Hospital-based controls	1, 2, 3, 4, 5, 6, 7
Eide et al. (8)	Norway	Case–control	440/43,387	70.7 ± 9.8/ 57.3 ± 12.9	220/440 (50.0%)	Sex	American-European Guideline (2005)	Population-based healthy controls	1, 2, 4
Eide et al. (9)	Norway	Case–control	176/35,413	61.2 ± 8.3/ 52.8 ± 9.6	95/176 (54.0%)	Sex	American-European Guideline (2005)	Population-based healthy controls	1, 2, 4
Jaraj et al. (10)	Sweden	Case–control	26/130	84.9 ± 4.0/ 84.9 ± 4.0	16/26 (61.5%)	Age, sex, cohort	American-European Guideline (2005)	Population-based healthy controls	1, 2, 4, 5, 6
Johansson et al. (11)	Sweden	Case–control	14/41	76.4 ± 5.1/ 70.5 ± 5.4	6/14 (42.9%)	Sex	American-European Guideline (2005)	Population-based healthy controls	1
Israelsson et al. (4)	Sweden	Case–control	176/368	74 ± 6/ 73 ± 6	73/176 (41.5%)	Age, sex	American-European Guideline (2005)	Population-based healthy controls, MMSE score ≥ 23	1–8
Ghaffari-Rafi et al. (13)	USA	Case–control	29/116	Median 83/ Median 57	N/A	Age, sex, race	American-European Guideline (2005)	Hospital-based control	1, 2, 4, 5, 7, 8
Rasanen et al. (14)	Finland	Case–control	60/49	76.9 ± 7.4/ 70.0 ± 8.4	32/60 (53.3%)	Sex	American-European Guideline (2005)	Asymptomatic relatives of the probable familial NPH patients that were ≥ 60 years old	1, 2, 4, 5, 7
Kuroda et al. (15)	Japan	Case–control	12/10	78.08 ± 8.43/ 76.6 ± 6.47	5/12 (41.7%)	Age, sex	Japanese Guideline (2012)	Hospital-based controls, MMSE-J score ≥ 28	1

1, Hypertension; 2, Diabetes Mellitus; 3, Hyperlipidemia; 4, Coronary heart disease; 5, Smoking; 6, Overweight; 7, Alcohol use; 8, Peripheral vascular disease; N/A, not applicable; MMSE-J, Japanese version of the Mini-Mental State Examination.

### 3.3. Association between vascular risk factors and iNPH

#### 3.3.1. Hypertension

**Figure 2:**
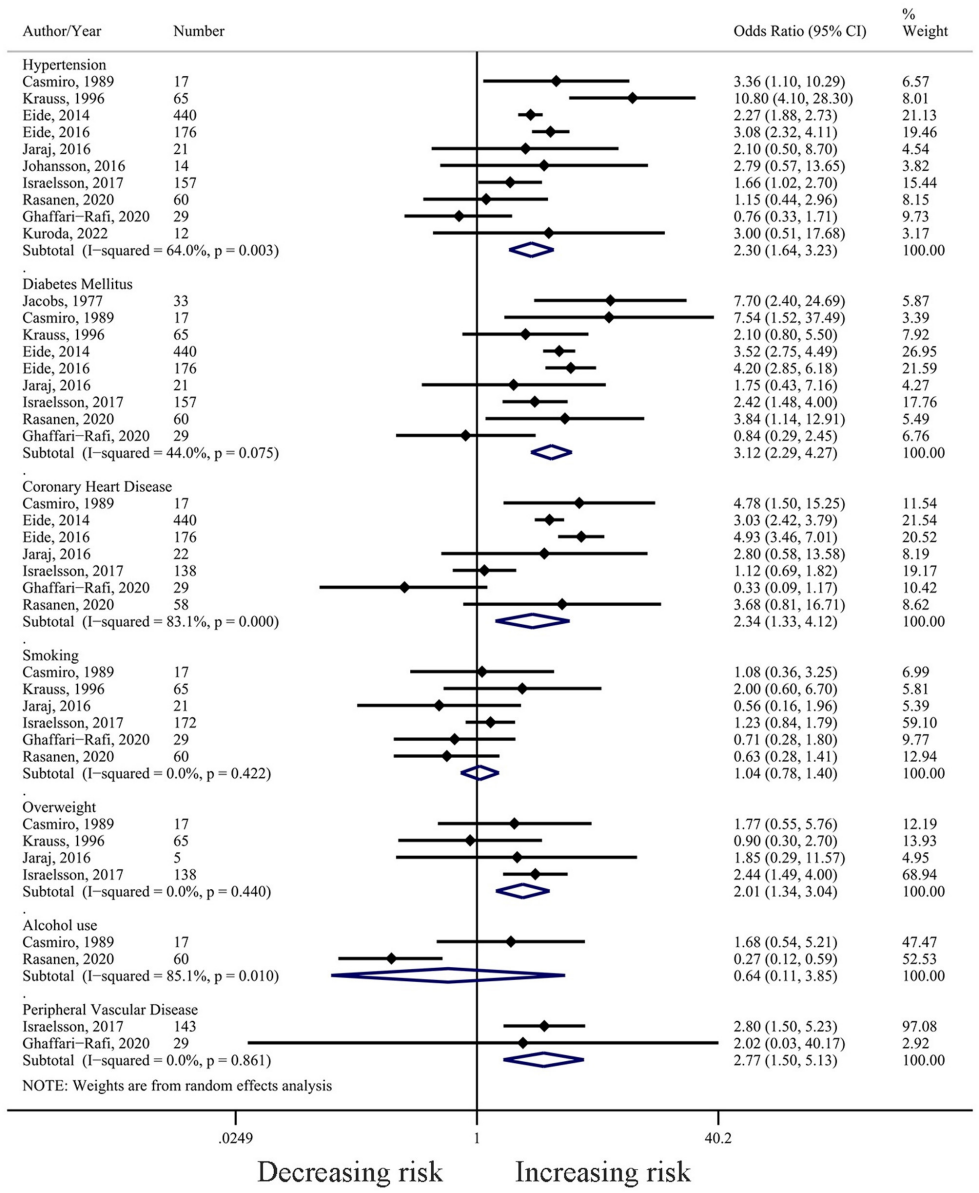
Forest plot (based on random-effect model) of the associations between hypertension, diabetes mellitus, smoking, alcohol use, overweight, coronary heart disease, and peripheral vascular disease with iNPH.

Hypertension was the most widely studied risk factor for iNPH, with 10 included studies (*N* = 991). The studies defined hypertension based on previous medical history, and in most (80%), the cut-off value was set at 140/90 mmHg, except for two early studies that used 160/95 mmHg and 160/90 mmHg (6, 7). The median proportion of hypertension was 65% (range: 40.91–85.71%). The results of meta-analysis showed that hypertension was associated with the diagnosis of iNPH (OR = 2.30, 95% CI 1.64 to 3.23, *I*^2^= 64.0%, *P* = 0.003) (Figure 2). Sensitivity analysis showed reliable and stable results (Supplementary Table S5A). The meta-regression analysis indicated that the year of publication might be the main source of heterogeneity in the results (Supplementary Table S6).

#### 3.3.2. Diabetes mellitus

Nine studies (*N* = 985) reported a relationship between DM and iNPH development. DM was mostly defined (89%) based on medical history or medication use, with only one study using the results of a glucose tolerance test (16). The median proportion of DM was 25.15% (Range: 13.04–51.52%). The combined result showed that DM was associated with the increasing risk for iNPH (OR = 3.12, 95% CI 2.29 to 4.27, *I*^2^= 44.0%, *P* = 0.075) (Figure 2). Sensitivity analysis revealed no effect on the stability of the results after using the leave-one-out method (Supplementary Table S4B). Meta-regression analysis showed that the age of the patients included in the study was the main source of heterogeneity in the results (Supplementary Table S5).

#### 3.3.3. CHD

In the included studies, CHD was defined as a previous diagnosis of CHD, angina pectoris, or myocardial infarction. The median proportion of CHD in the included studies was 19.32% (range: 13.8–47.1%). Pooled results from seven studies (*N* = 880) suggest that CHD may be a risk factor for the development of iNPH (OR = 2.34, 95% CI 1.33 to 4.12, *I*^2^= 83.1%, *P* = 0.000) (Figure 2). Sensitivity analysis confirmed the stability of the results (Supplementary Table S4C).

#### 3.3.4. Smoking

The combined results of the six studies (*N* = 364) suggest that smoking was not a risk factor for the development of iNPH (OR = 1.04, 95% CI 0.78 to 1.40, *I*^2^= 0.0%, *P* = 0.422) (Figure 2). Sensitivity analysis confirmed the stability of the results (Supplementary Table S4D).

#### 3.3.5. Overweight

A total of four studies (*N* = 225) discussed the relationship between overweight and the development of iNPH, and the results suggested that overweight was a risk factor for iNPH (OR = 2.01, 95% CI 1.34 to 3.04, *I*^2^= 0.0%, *P* = 0.440) (Figure 2). Two studies defined overweight as body mass index (BMI) ≧ 27 kg/m^2^ (6, 7), while another two defined it as BMI ≧ 25 kg/m^2^ (4, 10). Sensitivity analysis showed a significant change in the combined results of the remaining three papers after removing the study by Israelsson et al. (OR = 1.32, 95% CI 0.63 to 2.75, *I*^2^= 0.0%, *P* = 0.659) (Supplementary Table S4E).

#### 3.3.6. Hyperlipidemia

Three studies (*N* = 234) investigated the relationship between hyperlipidemia and iNPH and defined hyperlipidemia using different criteria. One study found that elevated Apolipoprotein B/A1 ratio was relevant with iNPH (OR = 2.51, 95% CI 1.61 to 3.9, *P* < 0.001) (4), while another two studies did not find a causal relationship between fasting triglyceride level (OR = 0.6, 95% CI 0.2 to 2.0, *P* = 0.37) or history of hyperlipidemia (OR = 0.90, 95% CI 0.40 to 2.04, *P* = 0.97) and iNPH (7, 13).

#### 3.3.7. Alcohol use

Only two studies (*N* = 77) examined the relationship between alcohol use and iNPH development, and the combined results showed that alcohol consumption was not a risk factor for iNPH (OR = 0.64, 95% CI 0.11 to 3.85, *I*^2^= 85.1%, *P* = 0.01) (Figure 2).

#### 3.3.8. Peripheral vascular disease

Two studies (*N* = 172) investigated the relationship between self-reported peripheral vascular disease and iNPH development and found that it was associated with the iNPH development (OR = 2.77, 95% CI 1.50 to 5.13, *I*^2^= 0.0%, *P* = 0.861) (Figure 2).

### 3.4. Publication bias

We visually inspected funnel plots and statistically used the Begg's and Egger's tests to evaluate publication bias for hypertension, DM, CHD, and smoking. As shown in funnel plots, no significant publication bias was detected (Supplementary Table S6, Supplementary Figure S1).

## 4. Discussion

In this study, we comprehensively reviewed the vascular risk factors for iNPH and identified five modifiable factors associated with iNPH. Hypertension was the most common vascular comorbidity, followed by DM, CHD, and peripheral vascular disease. Although overweight was also considered a potential vascular risk factor, its association with iNPH remained inconclusive owing to unstable sensitivity analysis results. In contrast, based on a few studies, smoking, alcohol consumption, and hyperlipidemia were not associated with iNPH.

The relationship between hypertension and iNPH has been reported in previous studies (4–8, 10, 12). Consistent with the previous literature, our study found that hypertension was the most common vascular risk factor in patients with iNPH. Meta-regression analysis found that the year of publication may be a source of heterogeneity, which could be explained by changes in the diagnostic criteria for hypertension (25). The association between arterial hypertension and iNPH was first described in 1987 (5), and a subsequent study found that only systolic blood pressure (BP) and pulse pressure, but not diastolic BP, were related to ventricular enlargement (26), which was in accordance with previous animal studies (27, 28). Hydrodynamic theory, a classic hypothesis of iNPH pathogenesis, may explain the association between hypertension and iNPH. Aging and hypertension can impair the elastic arteries' “Windkessel effect” (i.e., the ability of elastic arteries to distend during cardiac systole) (29). Consequently, a high pulse pressure is transmitted to the brain capillaries, leading to an increase in the pressure gradient within and outside the ventricles, eventually resulting in ventricular dilation (26, 30). Another animal study also demonstrated that arterial hypertension caused alterations in vessel dynamics that led to a decrease in perivascular pumping, subsequently reducing the overall cerebrospinal fluid flow within the perivascular spaces and further impacting the glymphatic system, a brain clearance pathway known to participate in the removal of amyloid-β (31, 32).

Several factors may explain the association between DM and iNPH. In mouse models, DM has been linked to neuroinflammation, waste accumulation, and reduced aquaporin 4 (AQP4) density, resulting in impairment of the glymphatic system (33, 34). This system was named after its similarity to the lymphatic system. AQP4 is the primary protein that facilitates material exchange (31). Recent studies have implicated reduced glymphatic clearance in iNPH development (35–37). Additionally, metabolic disturbances and microvascular damage resulting from DM may contribute to iNPH pathogenesis (11, 38). Another plausible explanation for the high proportion of DM comorbidities in patients with iNPH is that ventricular enlargement can cause mechanical stress-induced dysregulation of the hypothalamic-pituitary axis, resulting in dysregulation of hormonal secretion, as evidenced by decreased levels of growth hormone and insulin-like growth factor 1 in previous studies (39). However, age might act as a confounding factor in the relationship between DM and iNPH according to the meta-regression result. We cannot therefore exclude the possibility of spurious correlation between DM and iNPH in our study, given the inherent limitations of the included original studies. Larger cohort studies are warranted in the future to mitigate the influence of age on the conclusions.

Our meta-analysis suggested that overweight may be a risk factor for iNPH. Although no obvious heterogeneity was detected, instability in the results was found on omitting a large prospective case—control study (4). This may have resulted from multivariate adjustment of the effect size and different definitions of overweight across studies. Therefore, more consistent studies are needed to confirm the relationship between overweight and iNPH. There is limited knowledge regarding the underlying mechanism linking overweight to the development of iNPH, although a previous study revealed that a high BMI was associated with higher lumbar puncture opening pressure in patients with iNPH (40). Other research indicated that obesity was linked to decreased cerebral blood flow, alterations in gray matter, and microangiopathy (as observed by white matter hyperintensity and lacunar infarcts on magnetic resonance imaging) in healthy individuals (41–43). These findings may be due to the relationship between obesity and several pathophysiological changes, including neuroinflammation, mitochondrial dysfunction, and hormone alterations, which can exacerbate the process of neurodegeneration and cognitive decline (43–45). Further investigation is required to assess the potential risks of overweight in patients with iNPH.

In addition, we found that both CHD and peripheral vascular disease were risk factors for iNPH. However, heterogeneity may arise from variations in the diagnostic criteria across studies. Because these diseases are mainly atherosclerotic in nature, atherosclerosis may play a crucial role in the pathogenesis of iNPH (46). A previous autopsy-based study confirmed that severe hypertensive and arteriosclerotic vasculopathy with multiple lacunar infarcts was found in a patient with iNPH (47). Atherosclerosis causes ischemic-hypoxic damage to brain vessels and parenchyma, resulting in extensive changes in metabolism, blood-brain barrier function, and cerebrospinal fluid hydrodynamics (48). This contributes to demyelination and ventriculomegaly in patients with iNPH, as seen using magnetic resonance imaging (15). Further investigations are required to explore the pathophysiological mechanisms underlying these factors.

Given the high prevalence of vascular comorbidities among individuals with iNPH, it becomes imperative for clinicians to understand the potential impact of these factors on the surgical outcome. Vascular risk factors, such as hypertension, diabetes mellitus, coronary heart disease, peripheral vascular disease, cerebrovascular disease, and smoking, tend to exert a detrimental influence on the prognosis of iNPH patients (49–54). However, their effect on long-term outcomes appears to be relatively minor (51), albeit the lack of studies with extended follow-up periods. Consequently, the option of shunt surgery should not be denied, as nearly half of iNPH patients with cerebrovascular diseases still derive substantial benefits from shunt surgery over an extended duration (53, 54).

Our study had several limitations. First, most of the included studies were case-control studies, and establishing causality was challenging. Therefore, long-term follow-up studies are required. Second, some ORs were calculated from raw data without adjusting for potential confounders; we could not exclude unknown variables that affected the results. Third, publications written in languages other than English and conference proceedings were excluded, which might have resulted in a publication bias.

## 5. Implications for future studies

Further research is required to better understand the etiology and pathogenesis of iNPH. First, the current study had a cross-sectional design; therefore, we could not explore a causal relationship between these vascular risk factors and the development of iNPH. In addition, it is unclear whether these modifiable risk factors affect the prognosis of patients with iNPH. Long-term follow-up studies are needed to clarify these issues. Second, novel modifiable vascular risk factors, such as chronic kidney disease, physical inactivity, obstructive sleep apnea, inflammatory markers, and cerebral small vessel disease, also need to be explored to better understand disease mechanisms. Third, an internationally unified algorithm for diagnosing iNPH is required to compare different studies. Finally, despite conducting a systematic search, most studies were conducted in Western countries, and the current research lacks data from low- and middle-income countries; therefore, epidemiological characteristics and differences among these regions are urgently needed.

## 6. Conclusion

In this systematic review, we identified five modifiable vascular risk factors in patients with iNPH: Management of hypertension, DM, CHD, overweight, and peripheral vascular disease. These factors may be involved in the pathophysiological mechanisms promoting iNPH. Further studies are required to confirm these findings.

## Data availability statement

The original contributions presented in the study are included in the article/Supplementary material, further inquiries can be directed to the corresponding author.

## Author contributions

HC and FY conceived this study and drafted the manuscript. HC, FY, HG, KH, LQ, and RW collected the information and relevant materials. QC designed the study and revised the manuscript. All coauthors revised the manuscript and approved the final version.
