# Supplementary material for: Vascular risk factors for idiopathic normal pressure hydrocephalus: a systematic review and meta-analysis
Source: Front Neurol. 2023 Aug 10;14:1220473. doi: 10.3389/fneur.2023.1220473 (PMC10448702; doi:10.3389/fneur.2023.1220473)
Supplement: Supplementary file 1 [file Data_Sheet_1.docx]

Supplementary Material

**Vascular Risk Factors for Idiopathic Normal Pressure Hydrocephalus: A Systematic Review and Meta-analysis**

Hanlin Cai^1#^, Feng Yang^1#^, Hui Gao^1^, Keru Huang^2^, Linyuan Qin^1^, Ruihan Wang^1^, Yi Liu^2^, Liangxue Zhou^2^, Zilong Hao^1^, Dong Zhou^1^, Qin Chen^1*^

*** Correspondence:** Qin Chen, chen.qin@scu.edu.cn

**1 Supplementary Figures and Tables**

**1.1 Supplementary Tables**

**Supplementary-Table 1. Diagnostic criteria of iNPH in each study.**

|  | Jacobs, 1977 | Casmiro, 1989 | Krauss, 1996 | Eide, 2014 | Eide, 2016 | Jaraj, 2016 | Johansson, 2016 | Israelsson, 2017 | Ghaffari-Rafi, 2020 | Räsänen, 2020 | Kuroda, 2022 |
| --- | --- | --- | --- | --- | --- | --- | --- | --- | --- | --- | --- |
| Reference guideline | N/A | N/A | N/A | American-European Guideline (2005) | American-European Guideline (2005) | American-European Guideline (2005) | American-European Guideline (2005) | American-European Guideline (2005) | American-European Guideline (2005) | American-European Guideline (2005) | Japanese Guideline (2012) |
| Historical | N/A | N/A | N/A | N/A | N/A | History of severe head trauma, meningitis, or subarachnoid bleeding was used as exclusion criteria for suspected iNPH. | N/A | patient history | history of subarachnoid hemorrhage, trauma, structural lesions, previously compensated congenital hydrocephalus, malignancy (i.e., obstructive hydrocephalus), meningitis, stroke, transient ischemic attack, or in- tracerebral hemorrhage, were excluded | N/A | N/A |
| Clinical | At least one symptom of iNPH triad | At least one symptom of iNPH triad | Clinical presentation of NPH consisting of gait disturbance with or without dementia and/or urinary incontinence | 2–3 of the triad of gait ataxia, urinary incontinence and dementia | 2–3 of the triad of gait ataxia, urinary incontinence and dementia | gait disturbance according to examination or self-report, and either a Mini- Mental State Examination score of >=25 or urinary incontinence. | N/A | neurologic examination, | N/A | Based on questionaire | N/A |
| Radiological | hydrocephalic pneumoencephalography or radioisotopic cisternography findings | hydrocephalic CT findings | ventricular enlargement, and the absence of cortical atrophy. | increased ventricular size revealed by CT or MRI. | increased ventricular size revealed by CT or MRI. | radiologic findings of iNPH | N/A | MRI/CT revealing a com- municating hydrocephalus | N/A | N/A | all patients had dispropor- tionately enlarged subarachnoid space hydrocephalus on MRI |
| Physiological | N/A | N/A | N/A | Normal intracranial pressure (ICP) was documented by over-night ICP mon- itoring. | Normal intracranial pressure (ICP) was documented by over-night ICP mon- itoring. | N/A | N/A | CSF tap test or an infusion test | N/A | N/A | improvement in gait disturbance after the lumbar drainage test |
| Diagnosis | N/A | N/A | N/A | possible and probable iNPH | possible and probable iNPH | probable iNPH | possible and probable iNPH | probable iNPH | possible and probable iNPH | possible and probable iNPH | probable iNPH |

**Supplementary-Table 2. Definitions of vascular risk factors in each study.**

| **1. Hypertension** |  |  |  |  |  |  |  |  |
| --- | --- | --- | --- | --- | --- | --- | --- | --- |
| Author/Year | No. of pat. At risk | No. of pat. | Proportion | OR | 95%CI（Low） | 95%CI（Upper） | Statistic | Definition |
| Casmiro, 1989 | 11 | 17 | 64.71% | 3.36 | 1.1 | 10.29 | crude OR | Blood pressure values exceeding 160 and/or 95 mmHg on at least three different measurements,or treatment with anti-hypertensive drugs |
| Krauss, 1996 | 54 | 65 | 83.08% | 10.8 | 4.1 | 28.3 | adjusted OR | Documented history and treatment for hypertension, or blood pressure measures more than 160 mm Hg systolic and/or 90 diastolic on at least two different measurements. |
| Eide, 2014 | 184 | 440 | 41.82% | 2.27 | 1.88 | 2.73 | crude OR | Reported by the referring doctor/neurologist, and/or by the patient or his/her relatives |
| Eide, 2016 | 72 | 176 | 40.91% | 3.08 | 2.32 | 4.11 | crude OR | Reported by the referring doctor/neurologist, and/or by the patient or his/her relatives |
| Jaraj, 2016 | 9 | 21 | 42.86% | 2.1 | 0.5 | 8.7 | adjusted OR | Previous diagnosis of hypertension or use of antihypertensive medication. |
| Johansson, 2016 | 12 | 14 | 85.71% | 2.79 | 0.57 | 13.65 | crude OR | Either blood pressure > 140/90 or current use of blood pressure lowering medication |
| Israelsson, 2017 | 131 | 157 | 83.44% | 1.66 | 1.02 | 2.7 | Age- and sex-adjusted OR | Systolic blood pressure ≥140 mmHg or diastolic blood pressure ≥90 mmHg or use of anti-hypertensive drugs. |
| Rasanen, 2020 | 39 | 60 | 65.00% | 1.15 | 0.44 | 2.96 | Age-adjusted OR | Previous diagnosis of hypertension or use of antihypertensive medication. |
| Ghaffari-Rafi, 2020 | N/A | 29 | N/A | 0.76 | 0.33 | 1.71 | crude OR | The presence/absence of hypertension from electronic medical records |
| Kuroda, 2022 | 9 | 12 | 75.00% | 3 | 0.51 | 17.68 | crude OR | Presence of hypertension were detected based on self-reported medical history and/or medication use. |
|  |  |  |  |  |  |  |  |  |
| **2. Hyperlipidemia** |  |  |  |  |  |  |  |  |
| Author/Year | No. of pat. At risk | No. of pat. | Proportion | OR | 95%CI（Low） | 95%CI（Upper） | Statistic | Definition |
| Krauss, 1996 | 14 | 65 | 21.54% | 0.6 | 0.2 | 2 | adjusted OR | Fasting triglyceride level above 1.6 mmol/L |
| Israelsson, 2017 | 52 | 140 | 37.14% | 2.51 | 1.61 | 3.9 | Age- and sex-adjusted OR | ApoB/ApoA1 ratio >0.9 (male), >0.8 (female) |
| Ghaffari-Rafi, 2020 | N/A | 29 | N/A | 0.9 | 0.4 | 2 | crude OR | The presence/absence of hyperlipidemia from electronic medical records |
|  |  |  |  |  |  |  |  |  |
|  |  |  |  |  |  |  |  |  |
| **3. Diabetes Mellitus** |  |  |  |  |  |  |  |  |
| Author/Year | No. of pat. At risk | No. of pat. | Proportion | OR | 95%CI（Low） | 95%CI（Upper） | Statistic | Definition |
| Jacobs, 1977 | 17 | 33 | 51.52% | 7.7 | 2.4 | 24.69 | crude OR | Glucose tolerance test |
| Casmiro, 1989 | 4 | 17 | 23.53% | 7.54 | 1.52 | 37.49 | crude OR | Fasting blood glucose levels were determined;values exceeding upper normal limits (110mg/100ml) or treatment with hypoglycaemic drugs |
| Krauss, 1996 | 31 | 65 | 47.69% | 2.1 | 0.8 | 5.5 | adjusted OR | Documented history and treatment for diabetes, or fasting serum glucose levels above 6.7 mmol/L. |
| Eide, 2014 | 69 | 440 | 15.68% | 3.52 | 2.75 | 4.49 | crude OR | Reported by the referring doctor/neurologist, and/or by the patient or his/her relatives |
| Eide, 2016 | 26 | 176 | 14.77% | 4.2 | 2.85 | 6.18 | crude OR | Reported by the referring doctor/neurologist, and/or by the patient or his/her relatives |
| Jaraj, 2016 | 3 | 23 | 13.04% | 1.75 | 0.43 | 7.16 | crude OR | Previous diagnosis of DM type 1 or type 2, or pharmacologic treatment. |
| Israelsson, 2017 | 38 | 142 | 26.76% | 2.42 | 1.48 | 4 | Age- and sex-adjusted OR | History of treated diabetes mellitus or non-fasting p-glucose 11.1 mmol/l (198 mg/dl). |
| Rasanen, 2020 | 19 | 60 | 31.67% | 3.84 | 1.14 | 12.91 | Age-adjusted OR | Previous diagnosis of DM or pharmacologic treatment. |
| Ghaffari-Rafi, 2020 | N/A | 29 | N/A | 0.84 | 0.29 | 2.45 | crude OR | The presence/absence of type II diabetes mellitus from electronic medical records |
|  |  |  |  |  |  |  |  |  |
| **4. Overweight** |  |  |  |  |  |  |  |  |
| Author/Year | No. of pat. At risk | No. of pat. | Proportion | OR | 95%CI（Low） | 95%CI（Upper） | Statistic | Definition |
| Casmiro, 1989 | 6 | 17 | 35.29% | 1.77 | 0.55 | 5.76 | crude OR | BMI exceeding 27 |
| Krauss, 1996 | 19 | 65 | 29.23% | 0.9 | 0.3 | 2.7 | adjusted OR | BMI exceeding 27 |
| Jaraj, 2016 | 3 | 5 | 60.00% | 1.85 | 0.29 | 11.57 | crude OR | BMI exceeding 25 |
| Israelsson, 2017 | 113 | 138 | 81.88% | 2.44 | 1.49 | 4 | Age- and sex-adjusted OR | BMI exceeding 25 |
|  |  |  |  |  |  |  |  |  |
| **5. Smoking** |  |  |  |  |  |  |  |  |
| Author/Year | No. of pat. At risk | No. of pat. | Proportion | OR | 95%CI（Low） | 95%CI（Upper） | Statistic | Definition |
| Casmiro, 1989 | 9 | 17 | 52.94% | 1.08 | 0.36 | 3.25 | crude OR | Present or past smoking habits |
| Krauss, 1996 | 12 | 65 | 18.46% | 2 | 0.6 | 6.7 | adjusted OR | Previous or present habits of cigarette smoking |
| Jaraj, 2016 | 4 | 21 | 19.05% | 0.56 | 0.16 | 1.96 | crude OR | Past or present cigarette smoking |
| Israelsson, 2017 | 95 | 172 | 55.23% | 1.23 | 0.84 | 1.79 | Age- and sex-adjusted OR | Current smoker (smoking of any tobacco during the last year) or former smoker (quitted smoking more than a year earlier). |
| Ghaffari-Rafi, 2020 | N/A | 29 | N/A | 0.71 | 0.28 | 1.8 | crude OR | Current/former (smoked 100 or more cigarettes over lifetime) smoker |
| Rasanen, 2020 | 16 | 60 | 26.67% | 0.63 | 0.28 | 1.41 | crude OR | Past or present cigarette smoking |
|  |  |  |  |  |  |  |  |  |
| **6. Alcohol use** |  |  |  |  |  |  |  |  |
| Author/Year | No. of pat. At risk | No. of pat. | Proportion | OR | 95%CI（Low） | 95%CI（Upper） | Statistic | Definition |
| Casmiro, 1989 | 7 | 17 | 41.18% | 1.68 | 0.54 | 5.21 | crude OR | Daily consumption exceeding 50mg |
| Rasanen, 2020 | 19 | 60 | 31.67% | 0.27 | 0.12 | 0.59 | crude OR | Past or present alcohol use |
|  |  |  |  |  |  |  |  |  |
| **7. Coronary Heart Disease** |  |  |  |  |  |  |  |  |
| Author/Year | No. of pat. At risk | No. of pat. | Proportion | OR | 95%CI（Low） | 95%CI（Upper） | Statistic | Definition |
| Casmiro, 1989 | 8 | 17 | 47.06% | 4.78 | 1.5 | 15.25 | crude OR | Documented previous clinical admissions for myocardial ischaemic disease |
| Eide, 2014 | 90 | 440 | 20.45% | 3.03 | 2.42 | 3.79 | crude OR | Reported by the referring doctor/neurologist, and/or by the patient or his/her relatives |
| Eide, 2016 | 31 | 176 | 17.61% | 4.93 | 3.46 | 7.01 | crude OR | Reported by the referring doctor/neurologist, and/or by the patient or his/her relatives |
| Jaraj, 2016 | 4 | 22 | 18.18% | 2.8 | 0.58 | 13.58 | crude OR | Previous diagnosis of myocardial infarction or angina pectoris told by a physician. |
| Israelsson, 2017 | 32 | 138 | 23.19% | 1.12 | 0.69 | 1.82 | Age- and sex-adjusted OR | Previous myocardial infarction, left bundle blockage or angina pectoris. |
| Ghaffari-Rafi, 2020 | N/A | 29 | N/A | 0.33 | 0.09 | 1.17 | crude OR | The presence/absence of Coronary Artery Disease or Prior Myocardial Infarction from electronic medical records |
| Rasanen, 2020 | 8 | 58 | 13.79% | 3.68 | 0.81 | 16.71 | crude OR | Previous diagnosis of coronary artery disease |
|  |  |  |  |  |  |  |  |  |
| **8. Peripheral Vascular Disease** |  |  |  |  |  |  |  |  |
| Author/Year | No. of pat. At risk | No. of pat. | Proportion | OR | 95%CI（Low） | 95%CI（Upper） | Statistic | Definition |
| Israelsson, 2017 | 128 | 143 | 89.51% | 2.8 | 1.5 | 5.23 | Age- and sex-adjusted OR | Self reported stenosis of extra cranial cerebral arteries, claudicatio intermittens or renal dysfunction (GFR<90 or GFR<60 (chronic kidney disease, CKD)), calculated from serum creatinine, age and gender by the CKD-EPI equation. |
| Ghaffari-Rafi, 2020 | N/A | 29 | N/A | 2.02 | 0.03 | 40.17 | crude OR | The presence/absence of peripheral vascular disease from electronic medical records |

**Supplementary-Table 3. Quality assessment of case-control studies**

| **Case-control studies** | **Selection** | | | | **Comparability** | **Exposure** | | | **Scores** | **Quality(＜6 low, 6-7 moderate, 8-9 high)** |
| --- | --- | --- | --- | --- | --- | --- | --- | --- | --- | --- |
|  | Case definition | Case representativeness | Control selection | Control definition |  | Exposure ascertainment | Same method | Non-Response rate |  |  |
| Jacobs, 1977 | ☆ | ☆ |  |  | ☆ | ☆ | ☆ | ☆ | 6 | moderate |
| Casmiro, 1989 | ☆ | ☆ | ☆ | ☆ | ☆ | ☆ | ☆ | ☆ | 8 | high |
| Krauss, 1996 | ☆ | ☆ |  | ☆ | ☆☆ | ☆ | ☆ | ☆ | 8 | high |
| Eide, 2014 | ☆ | ☆ | ☆ | ☆ | ☆ | ☆ | ☆ | ☆ | 8 | high |
| Eide, 2016 | ☆ | ☆ | ☆ | ☆ | ☆ | ☆ | ☆ | ☆ | 8 | high |
| Jaraj, 2016 | ☆ | ☆ | ☆ | ☆ | ☆☆ | ☆ | ☆ | ☆ | 9 | high |
| Johansson, 2016 | ☆ | ☆ | ☆ | ☆ | ☆ | ☆ | ☆ | ☆ | 8 | high |
| Israelsson, 2017 | ☆ | ☆ | ☆ | ☆ | ☆☆ | ☆ | ☆ | ☆ | 9 | high |
| Ghaffari-Rafi, 2020 | ☆ | ☆ | ☆ | ☆ | ☆☆ | ☆ | ☆ | ☆ | 9 | high |
| Räsänen, 2020 | ☆ | ☆ |  | ☆ | ☆ | ☆ | ☆ | ☆ | 7 | moderate |
| Kuroda, 2022 | ☆ | ☆ |  | ☆ | ☆ | ☆ | ☆ | ☆ | 7 | moderate |

**Supplementary-Table 4. Results of sensitivity analysis for specific risk factors**

**Table S4a. Sensitivity analysis of association between hypertension and iNPH.**

| **Hypertension** | **OR** | **95%CI** | **I^2^** | ***P* value** |
| --- | --- | --- | --- | --- |
| Omitting Casmiro, 1989 | 2.356 | [2.045; 2.713] | 67.5% | < 0.0001 |
| Omitting Krauss, 1996 | 2.293 | [1.990; 2.642] | 47.9% | < 0.0001 |
| Omitting Eide, 2014 | 2.503 | [2.024; 3.097] | 67.4% | < 0.0001 |
| Omitting Eide, 2016 | 2.180 | [1.856; 2.560] | 61.5% | < 0.0001 |
| Omitting Jaraj, 2016 | 2.371 | [2.060; 2.730] | 68.0% | < 0.0001 |
| Omitting Johansson, 2016 | 2.366 | [2.055; 2.723] | 68.0% | < 0.0001 |
| Omitting Israelsson, 2017 | 2.446 | [2.113; 2.832] | 64.9% | < 0.0001 |
| Omitting Rasanen, 2020 | 2.407 | [2.089; 2.773] | 64.9% | < 0.0001 |
| Omitting Ghaffari-Rafi, 2020 | 2.451 | [2.126; 2.825] | 54.2% | < 0.0001 |
| Omitting Kuroda, 2022 | 2.365 | [2.055; 2.722] | 67.9% | < 0.0001 |

**Table S4b. Sensitivity analysis of association between diabetes mellitus and iNPH.**

| **Diabetes mellitus** | **OR** | **95%CI** | **I^2^** | ***P* value** |
| --- | --- | --- | --- | --- |
| Omitting Jacobs, 1977 | 3.284 | [2.743; 3.931] | 43.0% | < 0.0001 |
| Omitting Casmiro, 1989 | 3.316 | [2.772; 3.965] | 47.3% | < 0.0001 |
| Omitting Krauss, 1996 | 3.405 | [2.841; 4.080] | 47.6% | < 0.0001 |
| Omitting Eide, 2014 | 3.169 | [2.448; 4.103] | 49.8% | < 0.0001 |
| Omitting Eide, 2016 | 3.152 | [2.581; 3.851] | 44.5% | < 0.0001 |
| Omitting Jaraj, 2016 | 3.385 | [2.829; 4.049] | 48.0% | < 0.0001 |
| Omitting Israelsson, 2017 | 3.513 | [2.904; 4.250] | 43.5% | < 0.0001 |
| Omitting Rasanen, 2020 | 3.339 | [2.790; 3.997] | 50.8% | < 0.0001 |
| Omitting Ghaffari-Rafi, 2020 | 3.484 | [2.909; 4.173] | 8.4% | < 0.0001 |

**Table S4c. Sensitivity analysis of association between coronary heart disease and iNPH.**

| **Coronary heart disease** | **OR** | **95%CI** | **I^2^** | ***P* value** |
| --- | --- | --- | --- | --- |
| Omitting Casmiro, 1989 | 2.887 | [2.430; 3.431] | 85.6% | < 0.0001 |
| Omitting Eide, 2014 | 2.773 | [2.132; 3.607] | 85.8% | < 0.0001 |
| Omitting Eide, 2016 | 2.488 | [2.047; 3.023] | 79.5% | < 0.0001 |
| Omitting Jaraj, 2016 | 2.920 | [2.460; 3.467] | 85.9% | < 0.0001 |
| Omitting Israelsson, 2017 | 3.342 | [2.785; 4.010] | 72.7% | < 0.0001 |
| Omitting Ghaffari-Rafi, 2020 | 3.036 | [2.556; 3.606] | 79.3% | < 0.0001 |
| Omitting Rasanen, 2020 | 2.910 | [2.451; 2.456] | 85.9% | < 0.0001 |

**Table S4d. Sensitivity analysis of association between smoking and iNPH.**

| **Smoking** | **OR** | **95%CI** | **I^2^** | ***P* value** |
| --- | --- | --- | --- | --- |
| Omitting Casmiro, 1989 | 1.042 | [0.771; 1.409] | 19.1% | 0.7900 |
| Omitting Krauss, 1996 | 1.003 | [0.744; 1.354] | 0.0% | 0.9822 |
| Omitting Jaraj, 2016 | 1.082 | [0.803; 1.459] | 0.0% | 0.6046 |
| Omitting Israelsson, 2017 | 0.825 | [0.523; 1.299] | 0.0% | 0.4059 |
| Omitting Ghaffari-Rafi, 2020 | 1.089 | [0.802; 1.479] | 5.2% | 0.5851 |
| Omitting Rasanen, 2020 | 1.126 | [0.825; 1.538] | 0.0% | 0.4556 |

**Table S4e. Sensitivity analysis of association between overweight and iNPH.**

| **Overweight** | **OR** | **95%CI** | **I^2^** | ***P* value** |
| --- | --- | --- | --- | --- |
| Omitting Casmiro, 1989 | 2.051 | [1.324; 3.176] | 24.4% | 0.0013 |
| Omitting Krauss, 1996 | 2.295 | [1.475; 3.570] | 0.0% | 0.0002 |
| Omitting Jaraj, 2016 | 2.023 | [1.329; 3.081] | 25.7% | 0.0010 |
| Omitting Israelsson, 2017 | 1.316 | [0.631; 2.747] | 0.0% | 0.4641 |

**Supplementary-Table 5. Results of meta-regression for specific risk factors**

| Meta-regression | Covariant | No. of sources | Tau^2^ | Adjusted R^2^ (%) | 95% CI | *P* value |
| --- | --- | --- | --- | --- | --- | --- |
| *Hypertension* | Age | 10 | 0.1786 | 40.13 | 0.89, 1.01 | 0.110 |
|  | Sex | 9 | 0.215 | -33.28 | 0.48, 2.20 | 0.945 |
|  | Region | 10 | 0.2639 | 11.53 | 0.23, 1.93 | 0.412 |
|  | Year of publication | 10 | 0.0985 | 66.97 | 0.91, 0.99 | **0.031^*^** |
|  | Sample size | 10 | 0.3929 | -31.68 | 0.996, 1.004 | 0.971 |
| *Diabetes Mellitus* | Age  Sex | 9  8 | 0.0047  0.0199 | 93.17  <0.001 | 0.92, 0.99  0.50, 2.53 | **0.047^*^**  0.735 |
|  | Region | 9 | 0.0718 | -3.34 | 0.19, 2.71 | 0.576 |
|  | Year of publication | 9 | 0.0951 | -36.83 | 0.95, 1.01 | 0.192 |
|  | Sample size | 9 | 0.1344 | -93.44 | 0.998, 1.004 | 0.629 |
| *Coronary heart disease* | Age  Sex | 7  6 | 0.2102  0.3685 | 65.56  -22.92 | 0.84, 1.02  0.36, 3.11 | 0.091  0.884 |
|  | Region | 7 | 0.2998 | 50.87 | 0.01, 1.14 | 0.059 |
|  | Year of publication | 7 | 0.6003 | 1.64 | 0.87, 1.06 | 0.346 |
|  | Sample size | 7 | 0.7699 | -26.15 | 0.99, 1.01 | 0.665 |

Note: **^*^**Statistically significant.

**Supplementary-Table 6. Results of publication bias tests for specific**

**risk factors**

| Risk factors | No. of sources | Egger’s test | P value | Begg’s test | P value |
| --- | --- | --- | --- | --- | --- |
| Hypertension | 10 | -0.08 | 0.940 | 0.63 | 0.531 |
| Diabetes mellitus | 9 | -0.62 | 0.554 | 0.21 | 0.835 |
| Smoking | 6 | -0.93 | 0.404 | -0.19 | 0.851 |
| Coronary heart disease | 7 | -0.70 | 0.513 | -0.75 | 0.453 |

**1.2 Supplementary Figures**

**Supplementary-Figure 1.**

**Funnel plots for specific risk factors.**

**
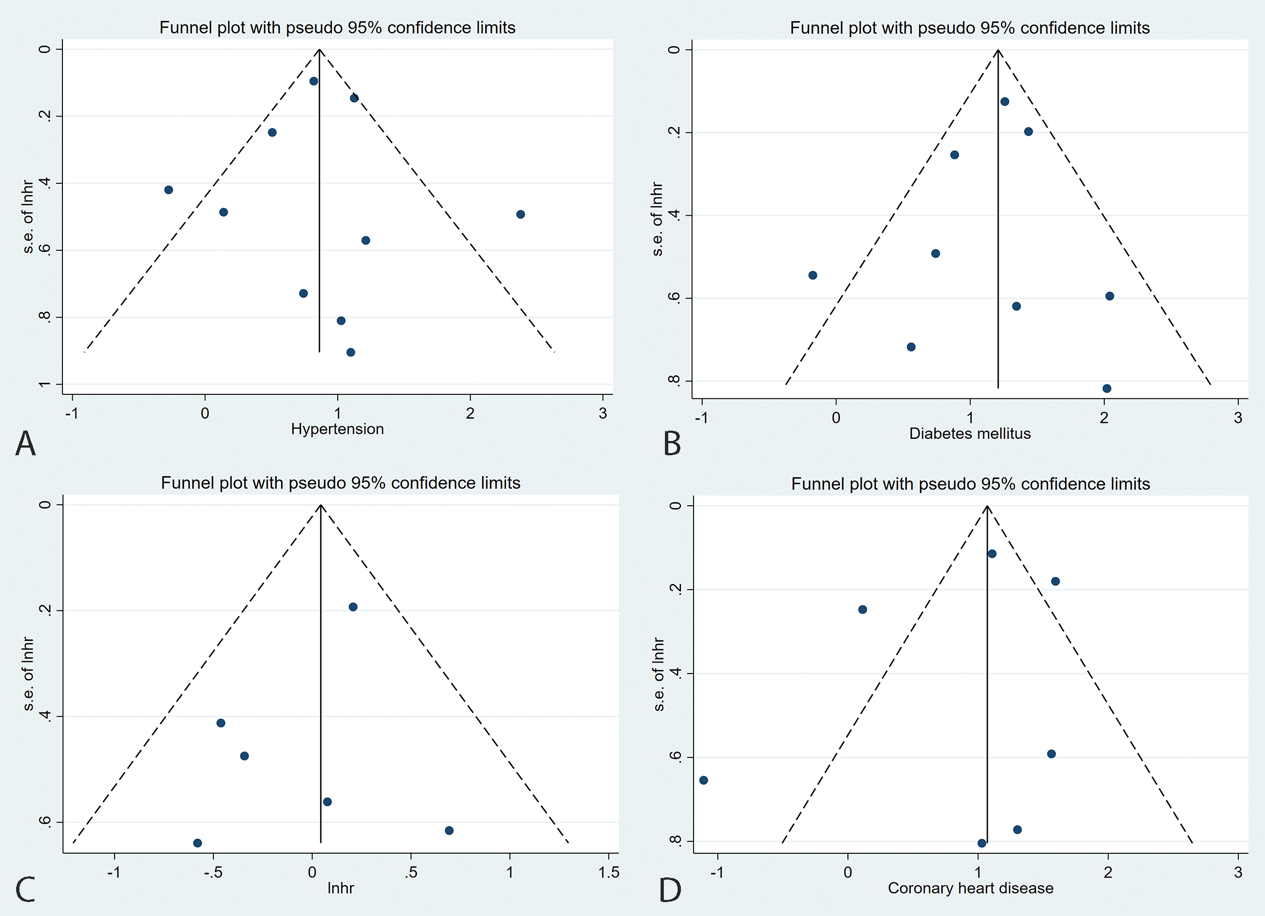
**

**Supplementary Figure 1.** Funnel plots of studies in hypertension(**A**), diabetes mellitus(**B**), smoking(**C**), and coronary heart disease(**D**).
